# Intratendinous Ganglionic Cyst of Semimembranosus: A Rare Cause of Thigh Swelling

**DOI:** 10.7759/cureus.20959

**Published:** 2022-01-05

**Authors:** Amman Yousaf, Shoaib Muhammad, Urooj Zahra, Fariha Ghaffar, Aribah Atiq, Nadin Elsayed, Muhammad S Ghaffar, Sara Yousaf, Ahmed Mounir Elsyaed, Syed Intekhab Alam

**Affiliations:** 1 Internal Medicine, McLaren Flint, Flint, USA; 2 Radiology, Services Hospital Lahore, Lahore, PAK; 3 Radiology, Salam Medical Complex, Lahore, PAK; 4 Department of Urology, Ghulab Devi Hospital, Al-Aleem Medical College, Lahore, PAK; 5 Internal Medicine, Fatima Jinnah Medical University, Lahore, PAK; 6 Internal Medicine, Allama Iqbal Medical College, Lahore, PAK; 7 Department of Pathology, Chughtai Laboratory, Lahore, PAK; 8 Department of Pathology, Shaukat Khanum Memorial Cancer Hospital and Research Centre, Lahore, PAK; 9 Diagnostic Radiology, Alexandria University, Alexandria, EGY; 10 Cardiothoracic Surgery, University of Pittsburgh, Pittsburgh, USA; 11 Anesthesiology and Critical Care, Kaul Associates, Lahore, Lahore, PAK; 12 Orthopaedics, Weill Cornell Medical College, Doha, QAT; 13 Orthopaedics Surgery, Hamad Medical Corporation, Doha, QAT; 14 Musculoskeletal Radiology, Hamad Medical Corporation, Doha, QAT

**Keywords:** ganglion, cyst excision, thigh mass, ganglionic, tendon cyst

## Abstract

Ganglionic cysts are common swellings of the hands. Various mechanisms are thought to generate these lesions, such as cystic mucoid degeneration and inflammation. Typically, ganglionic cysts are asymptomatic but can cause pain. They usually originate from soft tissues like ligaments, joint capsules, and sheaths of tendons. We present the case of a 37-year-old man with mid-thigh swelling with intermittent mild pain. However, no systemic symptoms like fever or weight loss were present. Workup unmasked the presence of a rare intratendinous ganglionic cyst. Ultrasonography (USG) and magnetic resonance imaging (MRI) can confirm the presence of ganglionic cysts and estimate their sizes and relationships with the surrounding structures. Treatment options range from observation and conservative management to interventions like aspiration and surgical excision.

## Introduction

Ganglionic cysts are tumor-like lesions of the soft tissues [1]. Many patients remain asymptomatic, while others present with symptoms related to the structure of origin. Based on our PubMed search, only around 27 cases have been reported, mostly involving hands, wrists, and feet. The presence of ganglionic cysts in tendons is a rare phenomenon. We present a 37-year-old male who presented with thigh swelling that was later diagnosed as an intra-tendinous ganglionic cyst of the semimembranosus muscle. To the best of our knowledge, this is the third case report presenting a ganglionic cyst in this rare location [2,3].

## Case presentation

A 37-year-old male patient presented to the outpatient clinic with swelling along the posterior-medial compartment of the right thigh for two months. He had a significant history of right foot trauma, extensor digitorum tendon rupture, and/or repair. The swelling was painless and gradually increased in size. He had no history of fever, weight loss, or anorexia. On examination, he was healthy-looking, and his vitals were within normal limits. Thigh examination revealed an oval-shaped swelling in the posterior-medial compartment of the distal thigh, almost 6 × 3 cm^2^ in size, with no overlying skin discoloration, redness, or punctum. There were no other swellings or enlarged lymph nodes.

**Figure 1:**
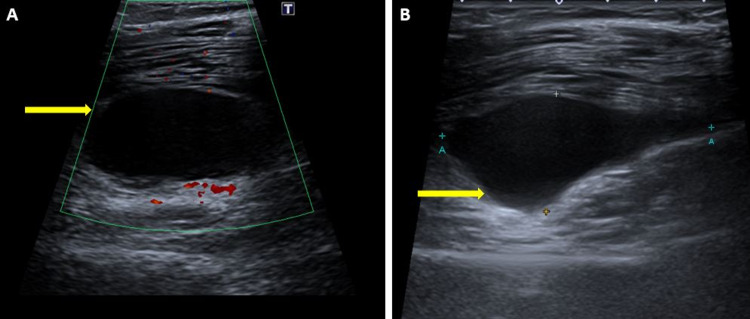
Ultrasound of the right thigh demonstrating a well-defined cystic lesion (yellow arrows) within the tendon of semimembranosus with no internal vascularity.

Ultrasound examination of the thigh revealed a well-defined cystic lesion within the tendon of the semimembranosus muscle measuring 5 × 3.6 cm^2^ with no internal vascularity (Figure 1).

The surrounding nerves and overlying subcutaneous planes were normal. The patient was reassured and magnetic resonance imaging (MRI) of the right lower leg was requested for further investigation. 

**Figure 2:**
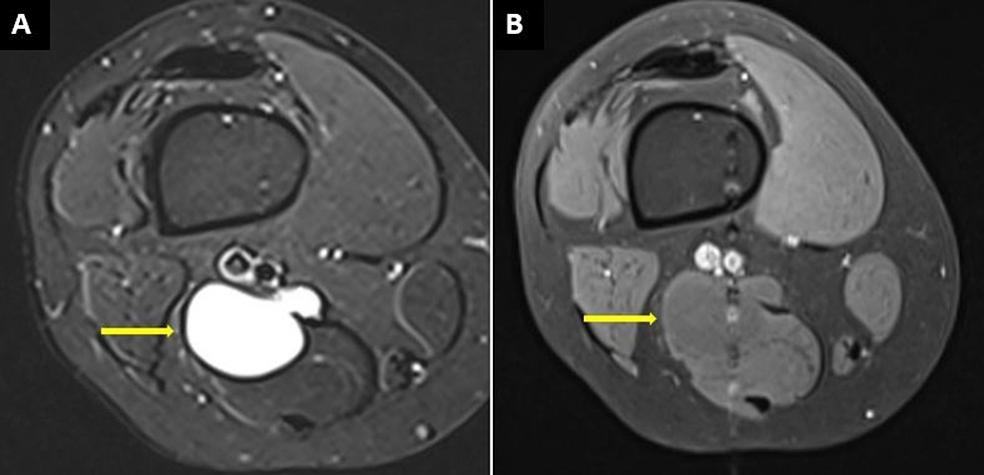
MRI of the right thigh (A) Axial-STIR sequence demonstrating a well-defined high signal intensity lesion originating from the semimembranosus tendon. (B) On the post-contrast sequence, there is minimal rim enhancement with no remarkable internal enhancement of the lesion.

**Figure 3:**
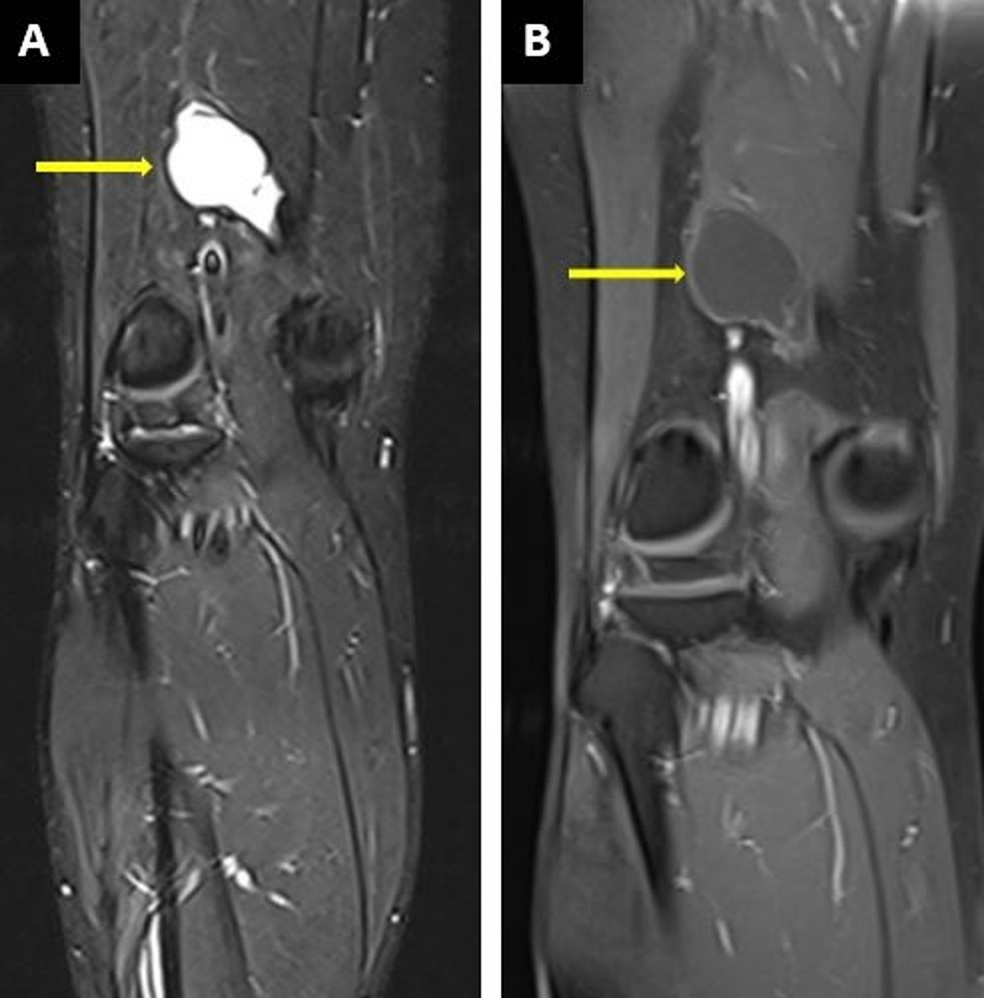
MRI of the right thigh (A) Coronal-STIR sequence demonstrating a well-defined high signal intensity lesion originating from the semimembranosus tendon. (B) On the post-contrast sequence, there is minimal rim enhancement with no remarkable internal enhancement of the lesion.

MRI revealed a well-defined cystic lesion along the semimembranosus tendon, 5 cm from superior to inferior and 3.6 cm medial to lateral in the lower part of the semimembranosus muscle and tendon. It showed a low T1 and bright T2 signal intensity pattern with no signal drop in fat suppression sequences and there was no contrast enhancement (Figures 2A-2B, 3A-3B).

**Figure 4:**
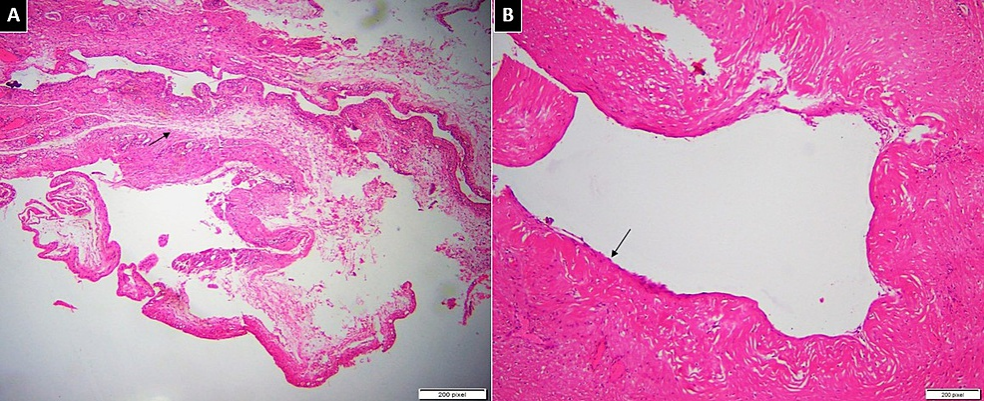
Histopathology of the excised specimen (A) Low power view of fibro collagenous cyst wall with no true epithelial lining. Few areas are showing foci of myxoid change (arrow). (B) High magnification shows dense collagenous wall (arrow) of ganglion cyst with no true lining. There is no nuclear atypia or mitotic activity.

These imaging findings were suggestive of an intra-tendinous ganglionic cyst. The patient was given conservative and surgical excision treatment options, and the chances of recurrence were explained. The patient decided to continue with conservative management and was offered analgesics as needed. However, a few weeks later, he started having pain, which worsened gradually. After six months, his pain reached a point where it was intolerable. In view of his severe and progressive symptoms, the surgical excision was made and the histopathology of the excised specimen was consistent with the ganglionic cyst (Figure 4A-4B).

On follow-up visits after three and six months, the patient remained asymptomatic and there is no recurrence to date.

## Discussion

Ganglionic cysts are mucin-filled cystic growths in the soft tissues. They usually arise from tendon sheaths, muscles, joint capsules, ligaments, and menisci. The cause of ganglionic cyst formation is still debatable, and various factors are thought to play a role. Mucinous degeneration due to recurrent injury of associated soft tissue is the most widely accepted mechanism of ganglionic cyst development [4]. Congenital factors are also believed to cause ganglionic cysts, especially in those with no history of injury or trauma to the associated soft tissues [5]. Other possible theories include nonspecific inflammation of the synovium causing hyperactive secretion. Additionally, tension and stretching of a joint capsule or a ligament can cause increased production of mucin. This fluid, in turn, forms the cysts by cutting through the ligament, sheath, or joint capsule [6].

The majority of ganglionic cysts (60-70%) arise from tendon sheaths and manifest as dorsal swellings on the wrist [6,7]. While the incidence of intra-tendinous ganglionic cysts is relatively rare and they typically present as wrist, hand, ankle, and foot swellings, our patient presented with a mid-thigh swelling [8]. The reported cases include ganglionic cysts in various tendons of the lower limbs, including the patellar tendon, quadriceps femoris tendon, and extensor longus tendon [1,8,9]. Ganglionic cysts are usually asymptomatic, with cosmetic problems as the only presenting complaints. This is particularly true in cases of hand and wrist lesions. However, if symptomatic, they can cause a variety of symptoms, from mild to severe pain, to affecting the mobility of surrounding structures. When joints are involved, cysts can interfere with normal functions of joints such as movement, snapping, locking, and catching [8].

Radiological modalities play a key role in diagnosing these cysts, especially when they are present in uncommon locations, as in our patient, leading to thigh muscle swelling. On ultrasonography (USG), ganglionic cysts appear as hypoechoic masses with multiple-sized lobules, septa, and acoustic enhancement [10]. USG can also estimate the size of the lesion and its relationship to the surrounding structures [1]. Typically, ganglionic lesions show no vascularity, which can be confirmed by colour Doppler USG. When the tail sign is positive, a diagnosis of the ganglionic cyst with significant confidence can be made. The tail sign is the presence of a thin neck connecting the cyst to the structure from where the lesion originates [8].

Magnetic resonance imaging can be used to confirm the lesion further and view its relation to the surrounding structures. On MRI, ganglionic cysts show thin enhancement in rim form with low signal intensity on T1 images, while images in the T2 phase show high signals. These features are demonstrated in the MRI findings of our case. It is important to note that if a solid component is seen within a cyst, a contrast-enhanced MRI should exclude any neoplastic lesion of the synovium [8]. 

Treatment options mainly depend on the location, size, and symptoms of the lesion. Cysts with no accompanying symptoms or cosmetic problems are generally managed conservatively with only symptomatic treatment. Initially, our patient was also treated conservatively for his mild pain; he was given an analgesic. Aspiration of fluid and surgical excision is the treatment choice for more extensive and symptomatic cysts. When our patient developed severe pain, he was managed with surgical resection. Recurrence is a significant drawback of aspiration (50%) and surgical excision (5%), and for this reason, most patients choose to follow observation and conservative management options [11].

## Conclusions

Intratendinous ganglionic cysts are a rare cause of soft tissue swelling. We suggest keeping this as a differential while investigating potential causes of musculoskeletal swellings. USG is an initial investigation that can confirm the presence of ganglionic cysts. MRI reveals a much more detailed image and a clearer relationship between the lesion and the surrounding structures. Both the imaging modalities hold excellent sensitivity and specificity for ganglionic cysts. Observation and conservative management are the mainstream treatment options. Patients with larger and symptomatic swellings can undergo aspiration and surgical excision of the cysts.
